# From printing to Scielo and Pubmed Central

**Published:** 2014-03-30

**Authors:** Luis Eduardo Bravo

**Affiliations:** 1 Director of the Cancer Registry of Cali. Department of Pathology, Universidad del Valle, Cali, Colombia.; 2 Editor-in-Chief (E) of Colombia Médica. Faculty of Health, Universidad del Valle, Cali, Colombia e-mail: luis.bravo@correounivalle.edu.co

The first issue of Philosophical Transactions from the Royal Society published in 1665 is widely regarded as the beginning of modern academic communication through journal articles. This form of presentation was an alternative to books for facilitating the exchange of ideas among scholarly circles. Soon journal collections and scientific libraries were created and eventually an evaluation of the quality of research activity was sought through noting the impact of their publications^1^.

Increased specialization, experimentation and an interdisciplinary approach to science dramatically increased academic productivity. In the last two centuries, the number of scientific articles has doubled every 10-15 years. From 1879-2004, in the National Library of Medicine^®^ of the United States (US-NLM^®^ is the English acronym) articles were indexed from medical journals in the *Index Medicus*, which went out of circulation due to the impossibility of managing the voluminous information generated in the form of a book^2^.

The eruption of digital and electronic formats and public use of the internet in the first half of the nineties changed the manner for disseminating scientific production. Commercial publishing houses proliferated and offered services that the scientific community and universities were unable to assume. Researchers freely ceded their copyrights and donated their writings to the publishers for the objective of having it published. Paradoxically, the university again paid for knowledge which it already owned and in some cases its own researchers had to pay the journals for its work to be published.

In response to the abuse by commercial publishing houses, the Open Access movement appeared as an alternative way for disseminating academic productions in which the journal guarantees free, irrevocable, unrestrictive access to the article from the date of its online publication. Reuse is regulated by Creative Commons (CC) licenses (http://creativecommons.org/about), the least restrictive among those adopted by publishers with open policies. Unfortunately, some traditional subscription journals created an open access system for some articles whose authors were paid a certain amount of money. This practice increases the appearance of predatory publishers of "Open-Access" that are more focused on making money than on spreading academic knowledge^3^.

A revolution also occurred over the past decade with the concept of biomedical libraries: changing them from centers of collection and organization of the medical literature (in book and magazine formats) to centers for the administration and management of information and medical knowledge. At the US-NLM significant changes were produced: the *Index Medicus *was transformed into MEDLINE^®^, a database created in the sixties that now offers over 20 million references to articles from over 5,600 biomedical and biological science journals published since 1946 and released worldwide. In 1996, Pubmed was created to manage this indexing system through a search engine that guarantees free access to online databases from the US-NLM, including MEDLINE^®^.

The dissemination of scientific information on the Internet requires prompt adoption of Web technologies by publishers to achieve a complete archive of the journals and ensure that the metadata used to describe the articles incorporates the best available technology. The full text of the journal and metadata should be encoded in a standardized manner to achieve interoperability among the different actors in global scientific productivity. The US-NLM in collaboration with Harvard University created a document type definition (DTD for its English acronym) for journal archives and later for use by publishers and authors. After the first users´ conference, the NISO-JATS standard (Journal Article Tag Suite, JATS for its English acronym) was adopted in 2011^4^.

This standard defines a set of elements and attributes in XML format for describing the full narrative content and metadata for a journal article where "journal article" has been broadly defined to include articles differing from those of research. This process has been essential for the consolidation of PubMed Central^®^ (PMC), a free archival system for complete texts of biomedical and biological science journals created by the US-NLM in 2000. PMC is the largest digital library in the world that serves as a digital counterpart to the extensive collections of printed journals in the US-NLM.

The transfer and implementation of these standards in Latin America has been possible thanks to BIREME, Brazil, the Specialized Center of PAHO/WHO that has fully adapted to the Internet as a production medium for sources and the flow of regional scientific and technical information. Since 1997, the current process began with the creation of SCIELO, a virtual library of scientific journals in electronic format which currently accommodates 967 journals from 12 Ibero-American countries. The library works with SCIELO methodology, a set of rules, guidelines, manuals and software for preparing, storing, disseminating and evaluating electronic publications. It organizes and publishes entire texts from online journals, plus producing and publishing indicators of its use and impact^5^.

Colombia Médica (CM) is at the top of the classification of the Colombian bibliographic index (Publindex) and has been accepted in several important global databases, including SciELO and HINARI. These achievements have been possible thanks to the dedication and generous efforts by the authors, peer reviewers and editorial groups led by Rodrigo Guerrero between 1970 and 1972; Francisco Falabella between 1973 and 1998; Pablo Barreto, Guillermo Llanos and Francisco Falabella from 1998 to 2001; Guillermo Llanos from 2002 to 2009 and Julian Herrera from 2010 to the present time. After 40 years of continuous effort and after complying with all quality standards, it entered the ISI citation index (Science Citation Index - Web of Science) in 2008 and MEDLINE^®^ in 2011. Print journals formalized its entry in MEDLINE^®^ with the periodic shipment of a copy of each issue of the journal to the US-NLM^6^.

For journals in an exclusively electronic format, such as CM, definitive indexing with MEDLINE^®^ is more demanding and costly because the journal must provide the US-NLM the complete content of the articles in the XML format with tags for bibliographic citations and a guarantee of access to all its content through a robust system that allows access and permanent preservation of its contents. In Latin America there are no certified digital repositories. The alternative is PMC, which in addition to ensuring free and unrestricted access, offers the archive free to journals that have their complete texts in English. 

Colombia Médica faces these demands with the institutional support of the Faculty of Health, Universidad del Valle, who determines the composition, functions and competencies of the Editorial and Scientific Committee of the journal, allowing the appointment of journal committees and internal calls for the appointment of Editor-in-Chief and Associate Editors^7^. The work team in turn is multidisciplinary, and includes proofing editors, translators, graphic designers, systems engineers, and technical and statistical consultants for labeling. These profound changes allow the publication of articles in English and the implementation of the Open Journal System to optimize the editorial workflow from the receipt of the manuscript to the publication of the article.

With guidance from the National University of Colombia and support from Scielo Brazil, in 2013 the standards of the US-NLM were implemented and technical endorsement by Pubmed Central^®^ was attained. Colombia Médica´s retention over time is guaranteed by the US-NLM; as of 2014 it will become part of PubMed Central^®^, the free digital archive of biomedical and biological sciences of the U.S. National Institute of Health, which is considered to be the most important digital library in the world.

The guidance of the journals by multidisciplinary teams, the policy of free access to its contents, and the technological transfer with the use of free software will help the journals of middle and low-income countries to overcome barriers to diffusion and to maintain technological independence from commercial publishers. Colombia Médica makes an impassioned plea to WHO/PAHO to support BIREME, Brazil and convert SciELO into a certified digital repository.
